# Optimum seeding density and seedling age for the outstanding yield performance of Japonica rice using crop straw boards for seedling cultivation

**DOI:** 10.3389/fpls.2024.1431687

**Published:** 2024-07-10

**Authors:** Yufei Ling, Qun Hu, Dihui Fu, Kaiwei Zhang, Zhipeng Xing, Hui Gao, Haiyan Wei, Hongcheng Zhang

**Affiliations:** ^1^ Key Laboratory of Crop Genetics and physiology of Jiangsu Province, Yangzhou University, Yangzhou, China; ^2^ Yangtze River Basin Rice Cultivation Technology Innovation Center of the Ministry of Agriculture of Yangzhou University, Yangzhou, China; ^3^ Collaborative Innovation Center of Modern Industrial Technology of Grain Crops, Yangzhou, China; ^4^ Research Institute of Rice Industrial Engineering Technology, Yangzhou, China

**Keywords:** rice, seeding density, seedling age, growth stage, yield formation, crop straw board

## Abstract

Crop straw boards, a novel nursery material, has proven effective for cultivating dense, young rice seedlings suitable for mechanized transplanting, thereby saving labor. However, under high-density nursery conditions, the biomass accumulation and yield formation in rice vary with different seedling ages, necessitating exploration of optimal seeding densities and seedling ages to achieve high yields. This study aims to determine the appropriate seeding densities and seedling ages using crop straw boards to maximize rice yield. Over two years, field studies were conducted using crop straw boards for rice cultivation at seeding densities of 150, 200, 250, 300, and 350 g/tray (labeled as D1, D2, D3, D4, and D5) and seedling ages of 10, 15, 20, and 25 days (labeled as A1, A2, A3, and A4).The results indicated that D4A2 significantly enhanced tiller number, dry matter accumulation, and photosynthetic capacity, resulting in a yield increase of 2.89% compared to the conventional method of D1A3. High-density and short-aged seedlings cultivated with crop straw boards can enhance rice yield by improving photosynthetic capacity and crop quality. This study emphasizes the importance of using crop straw boards for rice nursery practices, as well as selecting the appropriate seeding densities and seedling ages for optimizing rice production.

## 1 Introduction

Rice is the predominant staple crop in Asia, with about 60% of China’s population depending on it as their primary source of sustenance (Wu et al., 2013; Hu et al., 2018). With rising demands for rice and an aging demographic trend (Ren et al., 2021; Tang and Chen, 2022), achieving high rice yields is crucial for ensuring food security. Mechanical transplanting is well-regarded for its stability and high-yield potential (Zhang et al., 2014). However, the success of mechanical transplantation significantly depends on factors such as seeding density and seedling age, which affect growth, material accumulation, and thus overall field yield (Qiong et al., 2014; Li et al., 2020, 2021). Therefore, understanding the growth and development of rice seedlings and yield formation under various seeding densities and transplanting ages post-field transplanting is essential for effective rice field management and optimal yield achievement.

The conventional cultivation of japonica rice seedlings typically involves a seeding rate of 120-150 g/tray and transplantation at an age of 20-25 days (Liu et al., 2017). In response to the declining agricultural workforce, increasing the seeding rates presents a potential solution by reducing the number of trays required for transplantation and minimizing labor demand during cultivation and transplanting (Long et al., 2021; Ren et al., 2023). However, a higher seeding density can result in a reduction in the optimal age for transplantation, potentially leading to decreased yield if transplanted beyond the preferred seedling age (Li et al., 2014; Fei et al., 2015; Lampayan et al., 2015). Rice yield is heavily reliant on the number of tillers during the mature stage, which impacts leaf area and photosynthetic efficiency (Hao et al., 2017; Wang et al., 2018; Adachi et al., 2019; Silva et al., 2020). After rice heading, a higher leaf area index becomes crucial as it indicates a greater potential for converting dry matter into grain yield (Baloch et al., 2006; Fan et al., 2017). The findings of previous studies suggest that transplanting rice seedlings at an optimal age has minimal impact on rice tillering and dry matter accumulation. However, reducing the transplanting age can lead to increased tillering and dry matter accumulation, ultimately resulting in higher yields (Brar et al., 2012; Lampayan et al., 2019). This phenomenon is attributed to the vigorous activity and tillering capacity of rice seedlings under shorter seedling age transplant conditions (Xiong et al., 2009; Liu et al., 2015). However, due to the short age of seedlings, traditional rice seedlings exhibit a low lever of root entwining force and are unable to form a cohesive blanket-like structure, posing challenges for mechanical transplantation (Lin et al., 2015; Zhou et al., 2018). To address this issue, the utilization of crop straw boards is recommended due to their fiber-rich composition that aids in root connectivity. This approach enhances seeding density and strengthens root entwining force, thereby facilitating the cultivation of seedlings with reduced age suitable for mechanical transplantation (Ling et al., 2023).

This study specifically addresses the impact of varying seeding rates and seedling ages on the yield of japonica rice when using crop straw boards for seedling cultivation. Given the essential role of optimal seeding density and appropriate seedling age in maximizing yield, particularly in mechanical transplantation systems, we aim to determine the most effective strategies for rice cultivation under these conditions. The research focuses on understanding how different seeding densities and ages influence the physiological development of rice seedlings and their subsequent yield outcomes when integrated with crop straw board technology. By optimizing these variables, this study not only seeks to enhance yield and dry matter accumulation but also aims to provide actionable insights for field management, thus modernizing rice production and addressing workforce efficiency challenges. The outcomes of this investigation are expected to contribute significantly to theoretical and practical advancements in rice cultivation, emphasizing ‘light simplification’ and ‘reduced manpower requirements’, thereby laying the foundation for enhancing the efficiency of mechanized rice seedling transplantation.

## 2 Materials and methods

### 2.1 Experimental site and cultivars

The experiment was conducted at the off-campus base of Yangzhou University in Shiji Township, Sihong County, Jiangsu Province in 2021 and 2022(118°16’N, 33°22’E). The wheat was previously planted at this experimental site. At the initiation of the experiment, the topsoil horizon (0-20 cm) with a pH of 6.64 contained 23.52 g·kg^-1^ of organic matter, 1.34 g·kg^-1^ of total nitrogen, 16.34 mg·kg^-1^ of available phosphorus, and 127 mg·kg^-1^ of available potassium. Nanjing 5718 is a conventional medium-maturing japonica rice. As a high-quality and tasty rice from Jiangsu, Nanjing 5718 was selected as the tested variety in the experiment. Crop straw boards were provided by the Jiangsu Academy of Agricultural Sciences with a size of 57.5 cm × 27.5 cm × 2 cm.

### 2.2 Experimental design and management

Plastic seedling trays were used for cultivating rice seedlings. In this study, treatment consists of two parts: the first half represents the seeding density per tray, and the second half represents the seedling age. There are a total of 20 treatments (**Table 1**). The treatment’s name consists of two parts: the first half represents the seeding density per tray, and the second half represents the seedling age. The experiment for all treatments was repeated in triplicate. Then, these seedlings were transplanted to a paddy field on 18 June in 2021 and 2022. The Yangma high-speed transplanting machine (Model 2ZGQ-60D (G4) (YR60D)) was applied for transplantation. The planting density was 30 cm × 11.8 cm and 4 seedlings per hill for Nanjing 5718. The plot size for the field experiments was 316.8 m^2^ (7.2 m × 44 m), and the plots were also arranged in triplicate. The application of pure nitrogen was 270 kg·ha^-1^ in total, which was applied to the fields in the form of slow/controlled-release mixed fertilizer (Zhidaodi, Moith Corporation, China) one day before the seeding or the transplantation of seedlings. The element formula of the fertilizer can be expressed as N: P_2_O_5_: K_2_O=30:7:13. Water management and pest control were carried out in compliance with the prescribed local management protocols. For the convenience of illustration, seeds of 150, 200, 250, 300, and 350g in density at 3.20, 4.26, 5.33, 6.39, and 7.46 grains/cm² respectively are designated as D1, D2, D3 D4 and D5. Transplanted seedling age: Days 10, 15, 20, and 25, denoted as A1, A2, A3 and A4.

**Table 1:** Design of experimental treatments.

Seeding rate	Seed density	Seedling age	1000-grain weight	Seeding date	Transplant date
(g tray^-1^)	(Grains cm^-2^)	(d)	(g)	(m/d)	(m/d)
150	3.20	10	28.9	06/07	06/18
		15	28.9	06/02	06/18
		20	28.9	05/28	06/18
		25	28.9	05/23	06/18
200	4.26	10	28.9	06/07	06/18
		15	28.9	06/02	06/18
		20	28.9	05/28	06/18
		25	28.9	05/23	06/18
250	5.33	10	28.9	06/07	06/18
		15	28.9	06/02	06/18
		20	28.9	05/28	06/18
		25	28.9	05/23	06/18
300	6.39	10	28.9	06/07	06/18
		15	28.9	06/02	06/18
		20	28.9	05/28	06/18
		25	28.9	05/23	06/18
350	7.46	10	28.9	06/07	06/18
		15	28.9	06/02	06/18
		20	28.9	05/28	06/18
		25	28.9	05/23	06/18

### 2.3 Sampling and measurements

#### 2.3.1 Growth Stages Identification

Jointing Stage (JS): JS was determined when over 50% of rice plants had the first internode elongated over 1 cm, without adventitious roots in the upper internodes.

Heading Stage (HS): HS was marked by the emergence of the midsection of the young panicle from the flag leaf sheath, recognized when 50% of the plants displayed this trait.

Maturity Stage (MS): MS was indicated when over 95% of the panicles’ husks turned yellow, and grains became firm and translucent.

#### 2.3.2 Dry Matter Accumulation

At each identified growth stage (JS, HS, MS), the number of tillers was counted for 60 hills per plot. For the purpose of measuring dry matter, one intact plant was sampled from five representative hills, selected based on the average tiller count. Each part of the plant was oven-dried at 105°C for 30 minutes, followed by 80°C until a constant weight was obtained, enabling the calculation of dry matter accumulation per unit area.

#### 2.3.3 Leaf Area Measurement

Leaf area was quantified using a LI-3100 leaf area meter (LI-COR, USA). Samples were collected based on the arithmetic mean tiller number at each stage, with each sample categorized into leaves, stem-sheaths, and panicles. The leaf areas were specifically recorded for green leaves, and the effective leaf area index was calculated as the leaf area per unit area of effective tillers, emphasizing the top three leaves as the most effective leaf area.

#### 2.3.4 Yield Measurement During Maturity Stage

Before harvesting, the panicle count was conducted using the same methodology as the tiller count. During the maturity stage, all plants in a 2 m by 2 m area in the center of each plot were manually harvested. The grain yield was then measured and adjusted to a moisture content of 14.5%. The spikelet count per panicle, the percentage of filled grains, and the 1000-grain weight were determined from the harvested samples. Afterwards, the grains from each plot were dried at 35°C until their moisture content reached 14.5%.

### 2.4 Methods for calculating indicators


$$\begin{array}{c} \text{Decreasing rate of leaf area }\left(\text{LAI }{\text{d}}^{-1}\right)\\ =\left(\text{LA}{\text{I}}_{\text{MS}}-\text{LA}{\text{I}}_{\text{HS}}\right)/\text{Days from HS}-\text{MS} \end{array}$$

$$\text{Crop growth rate }\left(\text{g }{\text{m}}^{-2}{\text{d}}^{-1}\right)=\left({\text{W}}_{\text{MS}}-{\text{W}}_{\text{HS}}\right)/\text{Days from HS}-\text{MS}$$

$$\begin{array}{c} \text{Net assimilation rate }\left(\text{g }{\text{m}}^{-2}{\text{d}}^{-1}\right)\\ =[\left(\text{Ln }\left(\text{LA}{\text{I}}_{\text{MS}}\right)-\text{Ln }\left(\mathrm{LA}{\text{I}}_{\text{HS}}\right)/\left(\text{LA}{\text{I}}_{\text{MS}}-\text{LA}{\text{I}}_{\text{HS}}\right)\right]\\ \times \left[\left({\text{W}}_{\text{MS}}-{\text{W}}_{\text{HS}}\right)/\text{Days from HS}-\text{MS}\right] \end{array}$$

### 2.5 Statistical modeling

To analyze the influence of seeding density and seedling age on rice yield, a Gaussian function model was utilized, accounting for interactions between these two factors. The model is expressed as follows:


$$\begin{array}{c} z=z_{0}+B\exp\left\{-\frac{{\left(\ln\frac{x}{C}\right)}^{2}}{2D^{2}}\right\}+E\exp\left\{\frac{{\left(\ln\frac{y}{F}\right)}^{2}}{2G^{2}}\right\}\\ +H\exp\left\{\frac{{\left(\ln\frac{x}{C}\right)}^{2}}{2D^{2}}-\frac{{\left(\ln\frac{y}{F}\right)}^{2}}{2G^{2}}\right\} \end{array}$$

where x represents seeding density, y represents seedling age, and z is the estimated yield. The parametersz0​,B,C,D,E,F,G, and H encapsulate the model’s intercept and the effects of seeding density and seedling age, including their interactive effects.

### 2.6 Data calculation and statistical analysis

Excel 2016 (Microsoft Corporation, Redmond, WA, USA) was utilized for data processing. Statistical analyses were conducted using IBM SPSS Statistics 20.0 (IBM Corp., Armonk, NY, USA), a statistical analysis software, employed for the analysis of variance (ANOVA). The images were generated using Origin 2021 (OriginLab Corporation, Northampton, MA, USA).

## 3 Results

### 3.1 Leaf age and days of the regreening stage

**Figure 1:**
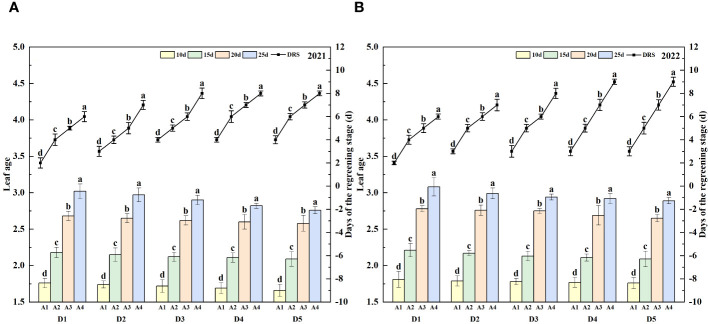
Leaf age and days of the regreening stage of rice affected by increased seeding density and seedling age with crop straw boards for seedling cultivation. **(A)** Leaf age and days of the regreening stage of rice in 2021. **(B)** Leaf age and days of the regreening stage of rice in 2022.

Under consistent seeding conditions, older rice seedlings exhibited prolonged leaf aging and required extended periods to regain their green coloration. Notably, in both 2021 and 2022, seedlings from treatment D1A4 demonstrated a regreening time that was 4 days longer than those from D1A1. Furthermore, with increased seeding densities, seedlings of equivalent age displayed reduced leaf aging, yet necessitated an additional 2 to 3 days for regreening, as depicted in **Figure 1**.

### 3.2 Growth stage

The growth stages of rice cultivation in paddy fields, when planted with young seedlings, consistently exhibited a delayed progression in both observed years, contrasting with fields containing older seedlings (**Tables 2**, **3**). As the rice plants continued to mature, this temporal gap gradually diminished. It is noteworthy that the field growth phase (FGP) for younger seedlings demonstrated an extended duration compared to their older counterparts. Importantly, throughout the whole growth phase (WGP), older seedlings remained in cultivation for an extended period of approximately 11-14 days longer than their younger counterparts. Furthermore, under controlled transplantation and uniform seedling age conditions, an increase in seeding density resulted in a shorter growth period while simultaneously prolonging the overall growth duration.

**Table 2:** Growth stages of rice affected by increased seeding density and seedling age with crop straw boards for seedling cultivation (2021).

Seeding density	Seedling age	ST	TS	JS	HS	MS	FGP	ST-JS	JS-HS	HS-MS	WGP
		(m/d)	(m/d)	(m/d)	(m/d)	(m/d)	(m/d)	(d)	(d)	(d)	(d)
D1	A1	06/07	06/18	08/02	09/02	10/30	134	56	31	58	145
	A2	06/02	06/18	08/02	09/02	10/29	133	61	31	57	149
	A3	05/28	06/18	08/01	09/01	10/28	132	65	31	57	153
	A4	05/23	06/18	07/31	08/31	10/27	131	69	31	57	157
D2	A1	06/07	06/18	08/02	09/02	10/30	134	56	31	58	145
	A2	06/02	06/18	08/02	09/02	10/30	134	61	31	58	150
	A3	05/28	06/18	08/01	09/01	10/29	133	65	31	58	154
	A4	05/23	06/18	07/31	08/31	10/28	132	69	31	58	158
D3	A1	06/07	06/18	08/02	09/02	10/30	134	56	31	58	145
	A2	06/02	06/18	08/02	09/02	10/30	134	61	31	58	150
	A3	05/28	06/18	08/01	09/01	10/29	133	65	31	58	154
	A4	05/23	06/18	08/01	09/01	10/29	133	70	31	58	159
D4	A1	06/07	06/18	08/03	09/03	11/01	136	57	31	59	147
	A2	06/02	06/18	08/03	09/03	10/31	135	62	31	58	151
	A3	05/28	06/18	08/02	09/02	10/30	134	66	31	58	155
	A4	05/23	06/18	08/01	09/01	10/29	133	70	31	58	159
D5	A1	06/07	06/18	08/04	09/04	11/02	137	58	31	59	148
	A2	06/02	06/18	08/04	09/04	11/01	136	63	31	58	152
	A3	05/28	06/18	08/03	09/03	10/31	135	67	31	58	156
	A4	05/23	06/18	08/02	09/02	10/30	134	71	31	58	160

ST, seeding time; TS, transplanting stage; JS, jointing stage; HS, heading stage; MS, maturing stage; FGP, field growth phase (from transplanting to harvesting); WGP, whole growth phase (from seeding in the seedbed to harvesting in the paddy field).

**Table 3:** Growth stages of rice affected by increased seeding density and seedling age with crop straw boards for seedling cultivation (2022).

Seeding density	Seedling age	ST	TS	JS	HS	MS	FGP	ST-JS	JS-HS	HS-MS	WGP
		(m/d)	(m/d)	(m/d)	(m/d)	(m/d)	(m/d)	(d)	(d)	(d)	(d)
D1	A1	06/07	06/18	08/04	09/01	11/03	138	58	28	63	149
	A2	06/02	06/18	08/04	09/01	11/02	137	63	28	62	153
	A3	05/28	06/18	08/03	08/31	11/01	136	67	28	62	157
	A4	05/23	06/18	08/02	08/29	10/30	134	71	27	62	160
D2	A1	06/07	06/18	08/04	09/01	11/03	138	58	28	63	149
	A2	06/02	06/18	08/04	09/01	11/03	138	63	28	63	154
	A3	05/28	06/18	08/03	08/31	11/02	137	67	28	63	158
	A4	05/23	06/18	08/02	08/29	10/31	135	71	27	63	161
D3	A1	06/07	06/18	08/04	09/01	11/04	139	58	28	64	150
	A2	06/02	06/18	08/04	09/01	11/04	139	63	28	64	155
	A3	05/28	06/18	08/03	08/31	11/03	138	67	28	64	159
	A4	05/23	06/18	08/02	08/30	11/02	137	71	28	64	163
D4	A1	06/07	06/18	08/04	09/02	11/05	140	58	29	64	151
	A2	06/02	06/18	08/04	09/02	11/05	140	63	29	64	156
	A3	05/28	06/18	08/04	09/01	11/04	139	68	28	64	160
	A4	05/23	06/18	08/03	08/31	11/03	138	72	28	64	164
D5	A1	06/07	06/18	08/04	09/03	11/06	141	58	30	64	152
	A2	06/02	06/18	08/04	09/02	11/05	140	63	29	64	156
	A3	05/28	06/18	08/04	09/01	11/04	139	68	28	64	160
	A4	05/23	06/18	08/03	08/31	11/03	138	72	28	64	164

ST, seeding time; TS, transplanting stage; JS, jointing stage; HS, heading stage; MS, maturing stage; FGP, field growth phase (from transplanting to harvesting); WGP, whole growth phase (from seeding in the seedbed to harvesting in the paddy field).

### 3.3 Number of stems and tillers

**Figure 2:**
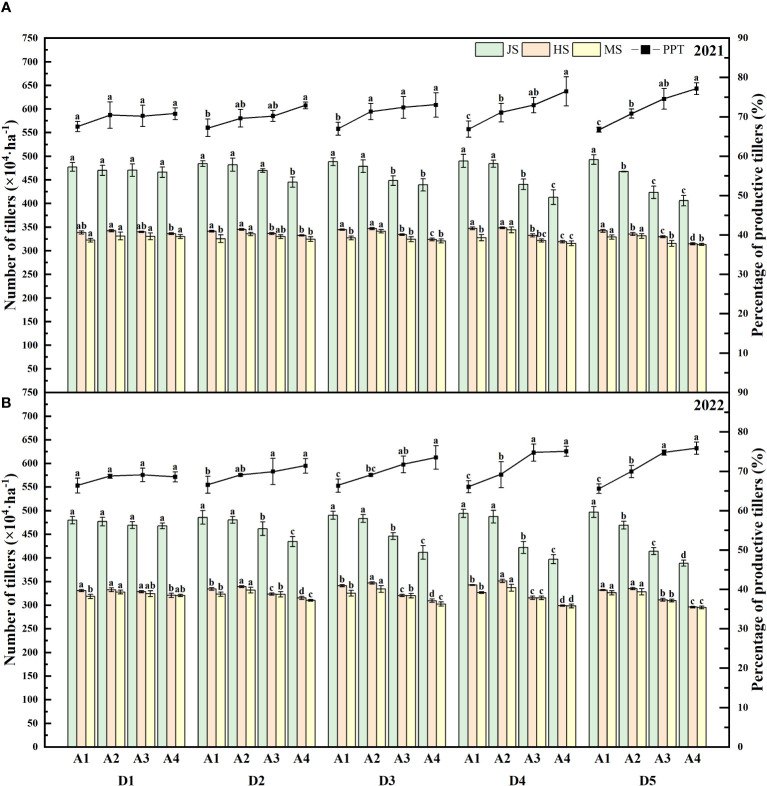
Tillers and percentage of productive tillers of rice affected by increased seeding density and seedling age with crop straw boards for seedling cultivation. **(A)** Tillers and percentage of productive tillers of rice in 2021. **(B)** Tillers and percentage of productive tillers of rice in 2022.

The investigation establishes a robust correlation between the age of transplanted seedlings and the percentage of productive tillers, with the effect becoming more evident at elevated seeding densities, as shown in **Figure 2**. For the years 2021 and 2022, D1A1 boasted a 4.02% and 2.64% higher proportion of productive tillers than D1A4, respectively. At the highest seeding density (D5), the variance further widened, with D5A1 leading D5A4 by 21.94% and 27.80%. Specifically, during the jointing stage of 2021, D5A1 had 487.98×10^4^·ha^-1^, slightly above D1A1’s 474.02×10^4^·ha^-1^. However, D5A4 displayed a significant decrease to 400.17×10^4^·ha^-1^, which is 17.99% lower than D5A1. The trend where the percentage of productive tillers initially increases with the age of the seedlings and then diminishes was observed, peaking with A2-aged seedlings across all seeding densities. Remarkably, the D4A2 treatment exhibited the highest percentages of productive tillers in both years, recording 344.18×10^4^·ha^-1^ and 336.97×10^4^·ha^-1^.

### 3.4 Leaf area index

**Tables 4** and **5** reveal the trends in the Leaf Area Index (LAI). During the Jointing Stage (JS), LAI gradually decreased with the increasing age of transplanted seedlings. For seeding densities between D1 and D4, LAI initially increased and then decreased with seedling age during the Heading Stage (HS) and Maturity Stage (MS). Notably, except for the D5 treatment, the highest LAI was consistently observed at seedling age A2 across all seeding density treatments during both the HS and MS stages. The 2021 data indicates that the peak LAI value for A2 seedlings at D4 seeding density was 7.14 during HS and 3.98 during MS, which were 19.60% and 40.64% higher, respectively, compared to D4A4 during the same periods.

**Table 4:** Leaf area index (LAI) affected by increased seeding density with crop straw boards for seedling Cultivation (2021).

Seeding density	Seedling age	JS	HS			MS
			Effective	High effective leaf area index		
		LAI	LAI	LAI	(%)	LAI
D1	A1	4.52a	6.89a	3.78a	54.96a	3.24ab
	A2	4.36ab	7.01a	3.83a	54.65a	3.38a
	A3	4.19bc	6.69ab	3.72ab	55.59a	3.18ab
	A4	4.02c	6.47b	3.61b	55.86a	3.04b
	**Mean**	**4.27a**	**6.77a**	**3.73a**	**55.27a**	**3.21b**
D2	A1	4.67a	6.93ab	3.87a	55.85a	3.38b
	A2	4.39ab	7.05a	3.98a	56.51a	3.63a
	A3	4.16bc	6.61bc	3.64b	55.11a	3.09c
	A4	3.98c	6.38c	3.53b	55.42a	2.96c
	**Mean**	**4.3a**	**6.74a**	**3.76a**	**55.72a**	**3.26ab**
D3	A1	4.71a	7.02a	3.91b	55.76a	3.58a
	A2	4.53a	7.12a	4.13a	57.99a	3.77a
	A3	3.89b	6.48b	3.57c	55.12a	3.03b
	A4	3.81b	6.11b	3.43d	56.17a	2.92b
	**Mean**	**4.23a**	**6.68ab**	**3.76a**	**56.26a**	**3.33a**
D4	A1	4.76a	7.08a	3.99b	56.44a	3.79b
	A2	4.61a	7.14a	4.17a	58.44a	3.98a
	A3	3.72b	6.29b	3.52c	55.99a	2.85c
	A4	3.23c	5.97b	3.37d	56.57a	2.83c
	**Mean**	**4.08b**	**6.62ab**	**3.76a**	**56.86a**	**3.36a**
D5	A1	4.83a	6.97a	3.82a	54.81a	3.64a
	A2	4.57a	7.01a	3.73a	53.23a	3.51a
	A3	3.61b	6.12b	3.44b	56.25a	2.79b
	A4	3.14c	5.89b	3.31b	56.27a	2.73b
	**Mean**	**4.04b**	**6.5b**	**3.58b**	**55.14a**	**3.17b**

TS, Transplanting stage; JS, Jointing stage; HS, Heading stage; MS, Maturity stage. LAI, Leaf area index. Different letters indicate statistical significance at the 0.05 probability level.The bold values represent the average values of all treatments under the same sowing density.

**Table 5:** Leaf area index (LAI) affected by increased seeding density with crop straw boards for seedling Cultivation (2022).

Seeding density	Seedling age	JS	HS			MS
			Effective	High effective leaf area		
		LAI	LAI	LAI	(%)	LAI
D1	A1	4.51a	6.81ab	3.71ab	54.49a	3.15ab
	A2	4.22b	6.99a	3.79a	54.26a	3.29a
	A3	4.15bc	6.77ab	3.66ab	54.09a	3.13ab
	A4	4.03c	6.52b	3.59b	55.11a	2.98b
	**Mean**	**4.23a**	**6.77a**	**3.69a**	**54.49a**	**3.14a**
D2	A1	4.55a	6.86a	3.74ab	54.56a	3.25ab
	A2	4.25b	7.06a	3.83a	54.30a	3.42a
	A3	4.09c	6.52b	3.62bc	55.55a	3.09bc
	A4	3.98c	6.33b	3.54c	56.03a	2.92c
	**Mean**	**4.22a**	**6.69a**	**3.68a**	**55.11a**	**3.17a**
D3	A1	4.59a	6.91a	3.79a	54.86a	3.33b
	A2	4.32b	7.14a	3.88a	54.35a	3.64a
	A3	3.93c	6.41b	3.53b	55.09a	3.04c
	A4	3.72d	6.17b	3.47b	56.24a	2.89c
	**Mean**	**4.14b**	**6.66a**	**3.67a**	**55.13a**	**3.22a**
D4	A1	4.64a	6.97a	3.82a	54.82a	3.36b
	A2	4.44b	7.25a	3.96a	54.64a	3.76a
	A3	3.78c	6.28b	3.48b	55.42a	2.95c
	A4	3.53d	5.94c	3.32b	55.91a	2.84c
	**Mean**	**4.1b**	**6.61a**	**3.64a**	**55.21a**	**3.23a**
D5	A1	4.73a	6.82a	3.77a	55.32a	3.26a
	A2	4.34b	6.93a	3.72a	53.69a	3.12a
	A3	3.67c	6.12b	3.41b	55.75a	2.84b
	A4	3.22d	5.81b	3.28b	56.45a	2.65c
	**Mean**	**3.99c**	**6.42b**	**3.55b**	**55.29a**	**2.97b**

TS, Transplanting stage; JS, Jointing stage; HS, Heading stage; MS, Maturity stage. LAI, Leaf area index. Different letters indicate statistical significance at the 0.05 probability level.The bold values represent the average values of all treatments under the same sowing density.

### 3.5 Crop growth indicators

**Figure 3:**
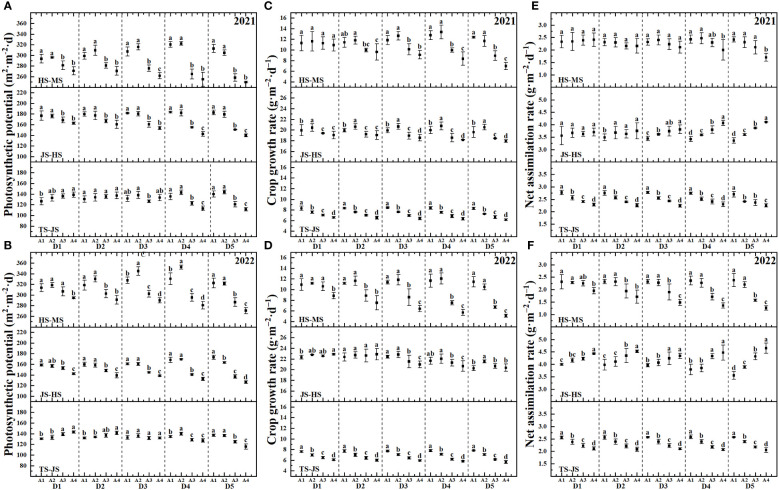
Crop growth indicators of rice affected by increased seeding density and seedling age with crop straw boards for seedling cultivation. **(A)** Photosynthetic potential of rice in 2021. **(B)** Photosynthetic potential of rice in 2022. **(C)** Crop growth rate of rice in 2021. **(D)** Crop growth rate of rice in 2022. **(E)** Net assimilation rate of rice in 2021. **(F)** Net assimilation rate of rice in 2022.

**Figure 3** indicates that during the Transplanting Stage (TS) to TS-JS, variations in rice photosynthetic potential were relatively minor. Under the same seeding rate, a declining trend was observed in the photosynthetic potential from JS-HS with increasing transplanting seedling age, which then showed a pattern of initial increase followed by a decrease during HS-MS. This trend became more pronounced with increased seeding density. For instance, in 2021, during the JS-HS period, the photosynthetic potential of D1A1 was 8.77% higher than D1A4, whereas D5A1 exceeded D5A4 by 30.73%; this changed to 8.39% and 25.26%, respectively, in the HS-MS period. The crop growth rate followed a similar pattern, with D1A1 exceeding D1A4 by 4.83% and D5A1 outperforming D5A4 by 9.50% during the JS-HS period, and 3.48% and 77.65%, respectively, during the HS-MS period. Notably, under high seeding densities, the net assimilation rate significantly increased with transplanting seedling age during the JS-HS period but decreased during the HS-MS period.

### 3.6 Biomass accumulation and ratio to total during the main stages

**Figure 4:**
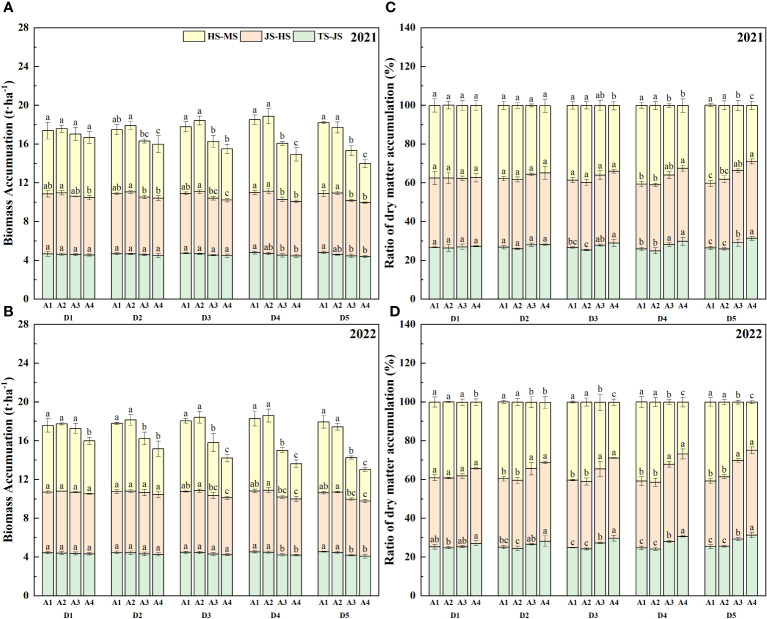
Biomass accumulation and ratio of dry matter accumulation of rice affected by increased seeding density and seedling age with crop straw boards for seedling cultivation. **(A)** Biomass accumulation of rice in 2021. **(B)** Biomass accumulation of rice in 2022. **(C)** Ratio of dry matter accumulation in 2021. **(D)** Ratio of dry matter accumulation in 2022.

The analysis of **Figure 4** indicates that during the JS, there was minimal variation in biomass accumulation among the treatments. However, in the MS, the D5 seeding density treatment showed a decline in biomass accumulation with increasing age of transplanted seedlings, while other seeding densities followed a pattern of an initial increase and then a decrease. Notably, under high seeding densities and older transplant ages, biomass accumulation during MS significantly dropped. For instance, in 2021 and 2022, biomass accumulation for D5A1 was 7.32 t·ha^-1^ and 7.16 t·ha^-1^, respectively, whereas D5A4 dropped to 4.05 t·ha^-1^ and 3.24 t·ha^-1^. Further research revealed that the ratio of dry matter accumulation in rice gradually diminished with the increase in seeding density and age of transplanted seedlings. This effect was particularly significant at seeding densities of D4 and D5, where the dry matter accumulation ratio at seedling age A4 was considerably lower compared to other treatments in similar conditions.

### 3.7 Dry matter weight per stem and population during the main stages

**Figure 5:**
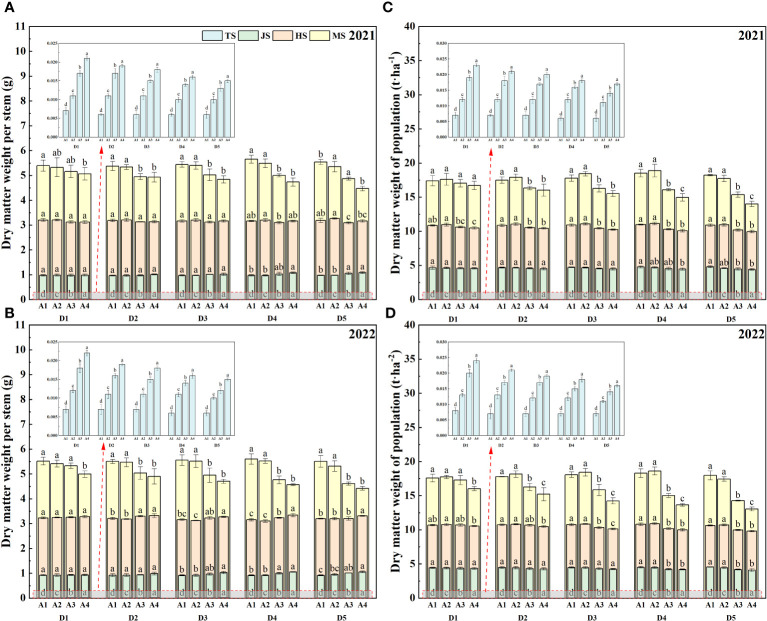
Dry matter weight per stem and dry matter weight of population of rice affected by increased seeding density and seedling age with crop straw boards for seedling cultivation. **(A)** Dry matter weight per stem of rice in 2021. **(B)** Dry matter weight per stem of rice in 2022. **(C)** Dry matter weight of population in 2021. **(D)** Dry matter weight of population in 2022.

During the TS, as evidenced by **Figure 5**, the dry matter weight per stem was at its peak for seedlings at age A4, and the total dry matter weight of the rice population also peaked at this stage. However, with increasing seeding densities, these values began to decline. During the JS and the HS, the differences between treatments were minimal. As shown in **Figure 3**, by the MS, an increase in seedling age and seeding density led to a reduction in dry matter weight, particularly noticeable at higher densities. For instance, in 2021 and 2022, the dry matter weight per stem for D1A1 was 3.31 g and 5.24 g higher, respectively, than for D5A4. Excluding the D5 density, with changes in seeding density, the population’s dry matter weight initially increased and then decreased, reaching the highest value at age A2. Specifically, as indicated by **Figure 3**, in 2021, D1A2 was 1.21% and 5.39% higher than D1A1 and D1A4, respectively, while D4A2 was 2.00% and 23.35% higher than D4A1 and D4A4.

### 3.8 Grain yield

The fitted parameters are presented along with their standard errors, which quantify the uncertainty associated with these estimates. The reduced chi-squared value of 0.05116 and the adjusted R-squared value of 0.87433 indicate a strong fit to the observed data. The impact of seedling age on rice yield was found to be less pronounced when transplanted within A1-A2, but a significant decrease in yield was observed as the seedling age increased to A3-A4. The treatments with D1-D4 exhibited a trend of increasing and then decreasing yields, with the highest yield observed when the transplanted seedling age was A2. Among these treatments, the highest yield was obtained in the D4A2 treatment. By optimizing the seeding density, age of transplanting seedlings, and the yield, it was determined that the high-density seeding model (D3-D5) can effectively maximize yield potential for A1 and A2 seedling ages.

**Figure 6:**
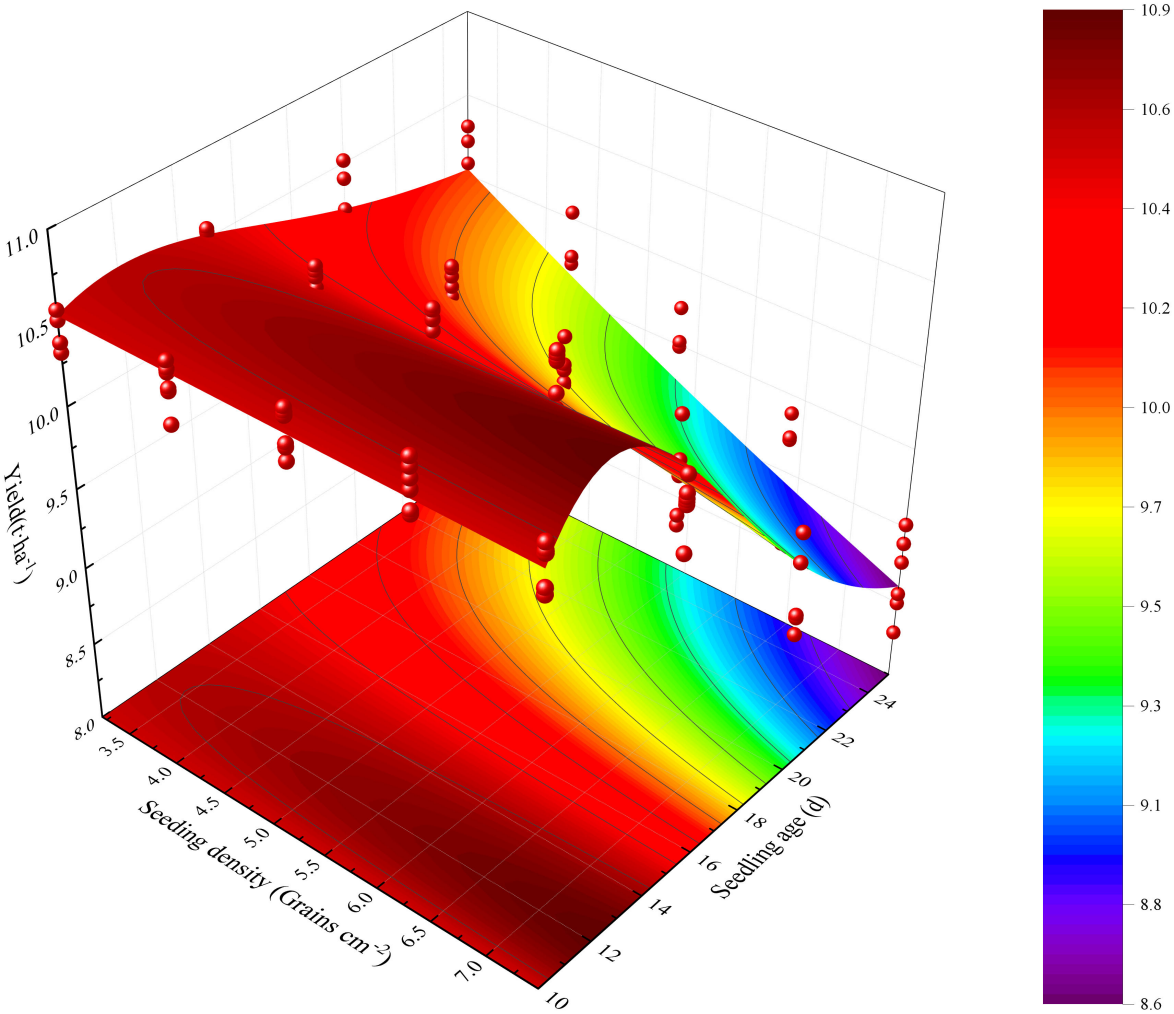
Rice yield affected by increased seeding density and seedling age with crop straw boards for seedling cultivation.

The maximum yield was observed in the D4A2 treatment, which was 2.89% higher than the conventional combination of D1A3. However, as the transplanting age of seedlings increases, there is a significant decline in yield. Conversely, with a conventional seeding density of D1, the impact of transplanting seedling age on rice yield is relatively minimal (**Figure 6**).

### 3.9 Correlation analysis

**Figure 7:**
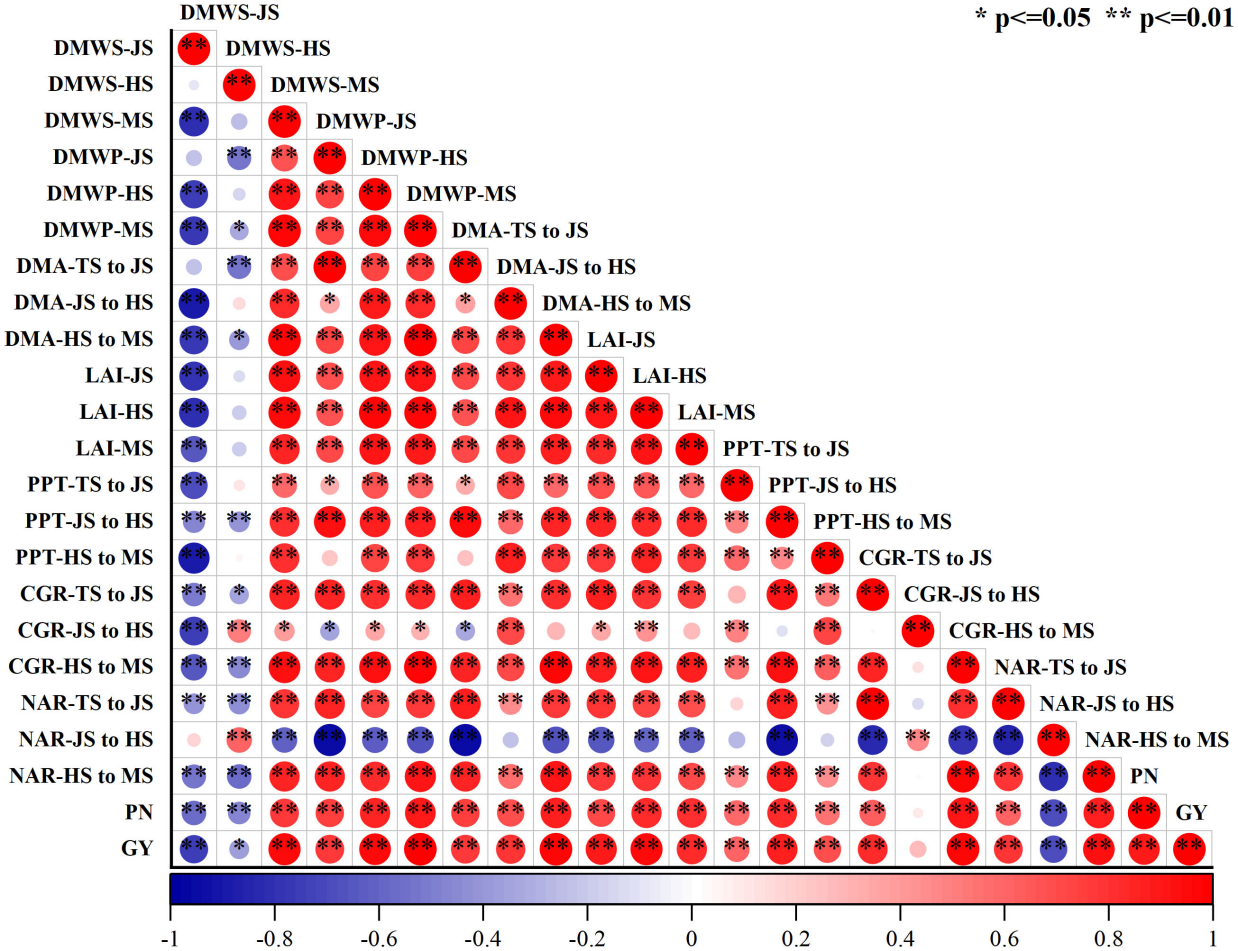
Correlation analysis of grain production and rice growth indicators affected by increased seeding density and seedling age with crop straw boards for seedling cultivation. (R 0.05 = 0.861 and R 0.01 = 0.895). DMWS-JS, Dry matter weight per stem during jointing stage; DMWS-HS, Dry matter weight per stem during heading stage; DMWS-MS, Dry matter weight per stem during mature stage; DMWP-JS, Dry matter weight of population during jointing stage; DMWP-HS, Dry matter weight of population during heading stage; DMWP-MS, Dry matter weight of population during mature stage; DMA-TS to JS, Dry matter accumulation from transplanting stage to jointing stage; DMA-JS to HS, Dry matter accumulation from jointing stage to heading stage; DMA-HS to MS, Dry matter accumulation from heading stage to mature stage; LAI-JS, Leaf area index during jointing stage; LAI-HS, Leaf area index during heading stage; LAI-MS, Leaf area index during mature stage; PPT-TS to JS, Photosynthetic potential from transplanting stage to jointing stage; PPT-JS to HS, Photosynthetic potential from jointing stage to heading stage; PPT-HS to MS, Photosynthetic potential from heading stage to mature stage; CGR-TS to JS, Crop growth rate from transplanting stage to jointing stage; CGR-JS to HS, Crop growth rate from jointing stage to heading stage; CGR-HS to MS, Crop growth rate from heading stage to mature stage; NAR-TS to JS, Net assimilation rate from transplanting stage to jointing stage; NAR-JS to HS, Net assimilation rate from jointing stage to heading stage; NAR-HS to MS, Net assimilation rate from heading stage to mature stage; PN, Panicle number; GY, Grain yield.

The correlation analysis revealed a positive association between all four aspects of yield components and grain yield. Specifically, grain yield exhibited a negative relationship with dry weight per stem during the joining stage and the net evaluation rate from jointing to heading stages. However, there was a significant positive correlation between grain yield and the net assessment rate from the heading stage to the maturity stage (**Figure 7**).

## 4 Discussion

The aging population and labor shortages in agriculture pose significant barriers to the traditional, labor-intensive methods of rice seedling cultivation and transplanting, thus impeding the adoption of mechanical techniques. The use of crop straw boards in high-density seedling cultivation reduces labor dependency and accelerates the formation of blanket seedlings, which are well-suited for mechanical transplanting. This research delves into the synergistic effects of seeding density and transplanting age on rice yield optimization.

### 4.1 Effects of seeding density and seedling age on the growth stage and tiller number of rice with crop straw board

Research indicates that the growth and regreening stages in rice are significantly influenced by leaf age (Ling et al., 1983; Jaffuel and Dauzat, 2005; Ye et al., 2008). While dense planting indeed exacerbates competition for resources such as light, water, and nutrients, thus affecting the regreening speed and growth of seedlings, other factors should also be considered, such as soil quality, moisture management, and root competition. Additionally, the impact of seeding density and seedling age on yield is pronounced, as higher seeding densities often lead to increased competition among seedlings, which can reduce overall yield unless managed with precise nutrient and water supplementation strategies. These factors can influence plant growth responses. For example, the space available for seedling root development may decrease with increasing planting density, further affecting root development and water use efficiency, thus impacting the overall health and growth rate of the plants (Shi et al., 2017; Lee et al., 2021). Reducing the age at which seedlings are transplanted can indeed accelerate their post-transplantation growth, primarily due to the higher growth vigor and adaptability of younger plants. However, very young seedlings might be more vulnerable during mechanical transplanting, more susceptible to damage from mechanical operations. The optimal transplanting age, which balances seedling robustness with growth potential, critically influences yield; younger seedlings, while quicker to establish, may not achieve their full yield potential due to insufficient root development and lower stress resilience. Additionally, the root systems of younger seedlings may not be sufficiently developed, which could impact their ability to establish themselves in new environments, especially in terms of water and nutrient absorption (Pasuquin et al., 2008; Li et al., 2017; Yang et al., 2019; Yuan et al., 2022; Ling et al., 2023). Older seedlings, with a higher leaf age at transplantation, experience about a 4-day delay in regreening and extend the overall growth phase by 11 to 14 days (**Figure 1**; **Tables 2**, **3**). Younger seedlings benefit from longer vegetative stages due to vigorous root activity that enhances root development post-transplantation and improves nutrient uptake, thus prolonging the vegetative phase. Additionally, previous studies have indicated that tiller numbers post-transplantation vary significantly with seeding densities and seedling ages, demonstrating that higher densities typically promote increased tillering, particularly when combined with optimal seedling ages that balance youthful vigor with resilience to transplanting stresses (Zhang et al., 2008; Cheng et al., 2019; Ling et al., 2023). For instance, during the 2021 jointing stage, D5A1 had a tiller count of 487.98×10^4^·ha^-1^, slightly higher than D1A1’s 474.02×10^4^·ha^-1^, while D5A4 only had 400.17×10^4^·ha^-1^, 17.99% less than D5A1 (**Figure 2**). Tiller numbers at maturity increase with seeding density, peaking with 15-day-old seedlings (**Figure 2**). Thus, selecting the appropriate transplanting age is critical for high-density cultivation to avoid premature or delayed transplanting, which aids in developing more effective tillers and an appropriate growth period.

### 4.2 Effects of seeding density and seedling age on the photosynthesis production and dry matter weight of rice with crop straw board

Leaf area significantly influences rice photosynthesis, crucial for plant growth and overall dry matter production. Increasing leaf area correlates positively with higher photosynthetic rates, which in turn boosts rice yield. Optimal seedling age and density ensure that leaf expansion maximizes photosynthesis without encountering diminishing returns from excessive crowding. Studies show that increasing leaf area within a reasonable range can enhance the photosynthetic capacity of rice, thereby increasing dry matter accumulation (Xiong et al., 2015; Tian et al., 2017). Leaf area in seedlings aged A1 to A4 expands initially then contracts, peaking at A2 (**Tables 4**, **5**). This dynamic change in leaf area affects yield, with peak leaf area at A2 corresponding to optimal photosynthetic efficiency and maximum yield potential. Extensive research has documented that prolonging the transplanting age from A2 to A4 leads to diminished stress resistance in seedlings, primarily attributed to the reduced enzymatic activities such as POD and CAT. These changes in enzymatic activity can delay the plant’s recovery from transplant shock, potentially reducing yield. A shorter recovery period facilitates quicker resumption of growth and can lead to higher overall yield. These enzymatic changes directly impact cellular processes critical for stress adaptation and recovery, thus affecting overall plant resilience and growth (Zhang et al., 2008; Liu et al., 2015; Shehata et al., 2022). This enzymatic reduction affects seedling quality, delays recovery, slows tillering, and ultimately reduces the leaf area index (Sui et al., 2013; Wang et al., 2019). As a result, under the same seeding density, an increase in seedling age is associated with a significant reduction in photosynthetic potential and growth rate during the HS and MS stages (**Figure 3**). To counter these effects, mechanically transplanted rice should be transplanted at the optimal age to achieve a larger leaf area, thereby enhancing photosynthetic capacity and laying the groundwork for increased biomass accumulation (Liu et al., 2017). Additionally, the accumulation of dry matter from the heading to maturity stages significantly dictates rice yield (Huang et al., 2019; Zhang et al., 2023). Under the same seeding density, dry matter weight trends upward initially then declines with seedling age during the HS-MS period (**Figures 4**, **5**), consistent with the trends observed in photosynthetic potential and growth rates (**Figure 3**). This is primarily because a higher leaf photosynthetic potential accelerates the conversion of carbon dioxide and water into carbohydrates such as glucose and synthesizes starch in the leaves to support the formation and development of grains (Takai et al., 2010; Gu et al., 2017). Our study indicates a positive correlation between dry matter weight and photosynthetic potential and growth rates (**Figure 7**). Thus, under high-density conditions, rice yield initially increases then decreases with transplanting age, with extended ages seeing more significant yield drops (**Figure 6**).

This study underscores the importance of optimizing the leaf area index (LAI) and seeding density to maximize photosynthesis and dry matter accumulation in rice, significantly impacting yield. It reveals that A2-aged seedlings at D4 density perform best from heading to maturity, highlighting the need for careful management of seedling age and density. Additionally, the use of crop straw boards as seedling material reduces labor for mechanical transplanting and enhances production efficiency. Further research is needed to explore the interaction between seeding density and transplanting age, and how they affect the occurrence of tillers at different leaf positions in rice. This will enable the analysis of the impact of tillers at different leaf positions on rice leaf area. Moreover, studying the mechanisms of photosynthate transfer across different densities and ages will contribute to enhancing rice yield and efficiency.

## 5 Conclusion

Increasing the seeding density in rice seedling cultivation prolongs the total growth phase by an estimated 3-4 days. Conversely, reducing the transplantation age can substantially shorten the growth phase by approximately 12-13 days. Compared to the traditional method of transplanting at 150 g/tray (3.2 grains·cm^-2^) after 20 days, an optimized approach using 300 g/tray (6.4 grains·cm^-2^) and transplanting at an earlier age of 15 days not only reduces the total growth phase but also significantly enhances dry matter accumulation, photosynthetic capacity, and yield. For instance, preliminary data suggest an increase in yield by up to 20% when using the optimized method. The use of crop straw boards for seedling cultivation further accentuates these benefits, possibly by improving root development and nutrient uptake. This study underscores the importance of optimizing seeding density and transplantation age to boost productivity, which could substantially decrease the costs associated with seedling cultivation. Furthermore, it lays a theoretical foundation for simplifying the production of mechanically transplanted rice in the future. To further explore high-yield rice models, future research should investigate the impact of different seeding densities on rice tillering number and quality, and how these factors influence overall plant health and yield.

## Data availability statement

The raw data supporting the conclusions of this article will be made available by the authors, without undue reservation.

## Author contributions

YL: Data curation, Investigation, Writing – original draft, Writing – review & editing. QH: Formal analysis, Methodology, Writing – original draft, Writing – review & editing. DF: Investigation, Writing – original draft. KZ: Investigation, Writing – original draft. ZX: Conceptualization, Investigation, Writing – original draft. HG: Conceptualization, Writing – original draft. HW: Conceptualization, Formal analysis, Writing – original draft. HZ: Conceptualization, Funding acquisition, Methodology, Resources, Supervision, Writing – review & editing, Writing – original draft.

## References

[B1] Adachi S. Yamamoto T. Nakae T. Yamashita M. Uchida M. Karimata R.. (2019). Genetic architecture of leaf photosynthesis in rice revealed by different types of reciprocal mapping populations. J. Exp. Bot. 70, 5131–5144. doi: 10.1093/jxb/erz303 31257428 PMC6793464

[B2] Baloch M. S. Awan I. U. Hassan G. (2006). Growth and yield of rice as affected by transplanting dates and seedlings per hill under high temperature of dera ismail khan, Pakistan. J. Zhejiang University.Science.B 7, 572–579. doi: 10.1631/jzus.2006.B0572 PMC1500883 16773732

[B3] Brar S. K. Mahal S. S. Brar A. S. Vashist K. K. Sharma N. Buttar G. S. (2012). Transplanting time and seedling age affect water productivity, rice yield and quality in north-west India. Agric. Water Manage. 115, 217–222. doi: 10.1016/j.agwat.2012.09.001

[B4] Cheng C. Lei K. Lu W. S. Huang S. H. Zhou Z. H. Gao B.. (2019). Effects of different seedling raising method and seedling age on seedling quality and grain yield of late japonica rice in southern China. Hybrid Rice 34, 46–51. doi: 10.16267/j.cnki.1005-3956.20190305.054

[B5] Fan Y. Tian Z. Yan Y. Hu C. Abid M. Jiang D.. (2017). Winter night-warming improves post-anthesis physiological activities and sink strength in relation to grain filling in winter wheat (triticum aestivum l.). Front. Plant Sci. 8. doi: 10.3389/fpls.2017.00992 PMC5469006 28659943

[B6] Fei T. Huizhe C. Defeng Z. Xueqing C. Jing X. Yicheng X.. (2015). Effects of sowing rates on seedling root entwining and seedling quality of machine-transplanted rice. Acta Agriculturae Universitatis Jiangxiensis 37, 398–403. doi: 10.13836/j.jjau.2015061

[B7] Gu J. Zhou Z. Li Z. Chen Y. Wang Z. Zhang H.. (2017). Photosynthetic properties and potentials for improvement of photosynthesis in pale green leaf rice under high light conditions. Front. Plant Sci. 8. doi: 10.3389/fpls.2017.01082 PMC5476740 28676818

[B8] Hao Z. Chao Y. Kewei C. Xiangsheng K. Hailang L. Junyi C.. (2017). Effect of direct-seeding methods on physiological characteristics and grain yield of rice and its cost analysis. Trans. Chin. Soc. Agric. Eng. 33, 58–64. doi: 10.11975/j.issn.1002-6819.2017.13.008

[B9] Hu Y. Wu P. Zhang H. Dai Q. Huo Z. Xu K.. (2018). Comparison of agronomic performance between inter-sub-specific hybrid and inbred japonica rice under different mechanical transplanting methods. J. Integr. Agric. 17, 806–816. doi: 10.1016/S2095-3119(17)61819-7

[B10] Huang M. Fan L. Jiang L. Yang S. Zou Y. (2019). Continuous applications of biochar to rice: effects on grain yield and yield attributes. J. Integr. Agric. 18, 563–570. doi: 10.1038/s41598-018-29877-7

[B11] Jaffuel S. Dauzat J. (2005). Synchronism of leaf and tiller emergence relative to position and to main stem development stage in a rice cultivar. Ann. Bot. 95, 401–412. doi: 10.1093/aob/mci043 15601682 PMC4246790

[B12] Lampayan R. Xangsayasane P. Bueno C. (2019). Crop performance and water productivity of transplanted rice as affected by seedling age and seedling density under alternate wetting and drying conditions in lao pdr. Water 11, 1816. doi: 10.3390/w11091816

[B13] Lampayan R. M. Faronilo J. E. Tuong T. P. Espiritu A. J. de Dios J. L. Bayot RS.. (2015). Effects of seedbed management and delayed transplanting of rice seedlings on crop performance, grain yield, and water productivity. Field Crops Res. 183, 303–314. doi: 10.1016/j.fcr.2015.08.014

[B14] Lee H. Hwang W. Jeong J. Yang S. Jeong N. Lee C.. (2021). Physiological causes of transplantation shock on rice growth inhibition and delayed heading. Sci. Rep. 11, 16818. doi: 10.1038/s41598-021-96009-z 34413345 PMC8376942

[B15] Li Y. Liu Y. Wang Y. Ding Y. Wang S. Liu Z.. (2020). Effects of seedling age on the growth stage and yield formation of hydroponically grown long-mat rice seedlings. J. Integr. Agric. 19, 1755–1767. doi: 10.1016/S2095-3119(19)62756-5

[B16] Li Y. Sun Y. Li Y. Lv T. Jiang M. Yan F.. (2017). Effects of mechanical-transplanted modes and density on root growth and characteristics of nitrogen utilization in hybrid rice at different seedling-ages. Chin. J. Rice Sci. 31, 599–610. doi: 10.16819/j.1001-7216.2017.7019

[B17] Li Z. Ma X. Xie J. Chen G. Zheng Z. Tan Y.. (2014). Experiment on precision seedling raising and mechanized transplanting of hybrid rice under low sowing rate in double cropping area. Trans. Chin. Soc. Agric. Eng. 30, 17–27. doi: 10.3969/j.issn.1002-6819.2014.06.003

[B18] Li Z. Zhou W. Zhang P. Zhong X. He L. Ren W.. (2021). Effect of seeding density and method on tillering characteristics of mechanical transplanting in indica ice. J. Nucl. Agric. Sci. 35, 722–736. doi: 10.11869/j.issn.100-8551.2021.03.0722

[B19] Lin Y. Zhang J. Hu Z. Zhu L. Yu S. Jin Q. (2015). Research on rice mechanized seedling substrate in China. China Rice 21, 7–13. doi: 10.3969/j.issn.1006-8082.2015.04.002

[B20] Ling Q. Su Z. Zhang H. Cai J. He J. (1983). The leaf age model of development process in different various of rice. Scientia Agricultura Sin. 24, 9–18. doi: 10.3864/j.issn.0578-1752.1983-16-01-9-18

[B21] Ling Y. Liu M. Feng Y. Xing Z. Gao H. Wei H.. (2023). Effects of increased seeding density on seedling characteristics, mechanical transplantation quality, and yields of rice with crop straw boards for seedling cultivation. J. Integr. Agric. doi: 10.1016/j.jia.2023.12.018

[B22] Liu Q. Wu X. Ma J. Chen B. Xin C. (2015). Effects of delaying transplanting on agronomic traits and grain yield of rice under mechanical transplantation pattern. PloS One 10, e0123330. doi: 10.1371/journal.pone.0123330 25875607 PMC4395310

[B23] Liu Q. Zhou X. Li J. Xin C. (2017). Effects of seedling age and cultivation density on agronomic characteristics and grain yield of mechanically transplanted rice. Sci. Rep. 7, 14072. doi: 10.1038/s41598-017-14672-7 29074876 PMC5658416

[B24] Long R. Leng S. Zhao L. Yin J. Yang J. Li G.. (2021). Preliminary study on mechanical transplanting technique of small indica rice seedlings in yunnan province. China Rice 27, 134–136. doi: 10.3969/j.issn.1006-8082.2021.05.029

[B25] Pasuquin E. Lafarge T. Tubana B. (2008). Transplanting young seedlings in irrigated rice fields: early and high tiller production enhanced grain yield. Field Crops Res. 105, 141–155. doi: 10.1016/j.fcr.2007.09.001

[B26] Qiong L. Kun W. Jingbo X. Guanghui C. (2014). Research progress on suitable seedling age of mechanical transplanting rice. Crops 30, 5–8. doi: 10.16035/j.issn.1001-7283.2014.05.002

[B27] Ren C. Zhou X. Wang C. Guo Y. Diao Y. Shen S.. (2023). Ageing threatens sustainability of smallholder farming in China. Nature 616, 96–103. doi: 10.1038/s41586-023-05738-w 36813965

[B28] Ren M. Huang M. Qiu H. Chun Y. Li L. Kumar A.. (2021). Genome-wide association study of the genetic basis of effective tiller number in rice. Rice (New York N.Y.) 14, 56. doi: 10.1186/s12284-021-00495-8 34170442 PMC8233439

[B29] Shehata S. A. Omar H. S. Elfaidy A. El-Sayed S. Abuarab M. E. Abdeldaym E. A. (2022). Grafting enhances drought tolerance by regulating stress-responsive gene expression and antioxidant enzyme activities in cucumbers. BMC Plant Biol. 22, 408. doi: 10.1186/s12870-022-03791-7 35987604 PMC9392319

[B30] Shi H. Zhu D. Zhang Y. Xiang J. Zhang Y. Zhu C.. (2017). Effects of biodegradable seedling tray and sowing rate on seedling quality and yield of mechanical transplanting rice. Trans. Chin. Soc. Agric. Eng. 33, 27–34. doi: 10.11975/j.issn.1002-6819.2017.24.004

[B31] Silva R. Filgueiras L. Santos B. Coelho M. Silva M. Estrada-Bonilla G.. (2020). Gluconacetobacter diazotrophicus changes the molecular mechanisms of root development in oryza sativa l. Growing under water stress. Int. J. Mol. Sci. 21, 333. doi: 10.3390/ijms21010333 31947822 PMC6981854

[B32] Sui B. Feng X. Tian G. Hu X. Shen Q. Guo S. (2013). Optimizing nitrogen supply increases rice yield and nitrogen use efficiency by regulating yield formation factors. Field Crops Res. 150, 99–107. doi: 10.1016/j.fcr.2013.06.012

[B33] Takai T. Kondo M. Yano M. Yamamoto T. (2010). A quantitative trait locus for chlorophyll content and its association with leaf photosynthesis in rice. Rice 3, 172–180. doi: 10.1007/s12284-010-9047-6

[B34] Tang R. D. Chen C. (2022). Effects of outsourcing services on elderly farmers participation in rice production. Chin. J. Rice Sci. 36, 647–655. doi: 10.16819/j.1001-7216.2022.220704

[B35] Tian G. Gao L. Kong Y. Hu X. Xie K. Zhang R.. (2017). Improving rice population productivity by reducing nitrogen rate and increasing plant density. PloS One 12, e0182310. doi: 10.1371/journal.pone.0182310 28767723 PMC5540556

[B36] Wang J. Lu K. Nie H. Zeng Q. Wu B. Qian J.. (2018). Rice nitrate transporter osnpf7.2 positively regulates tiller number and grain yield. Rice 11, 12. doi: 10.1186/s12284-018-0205-6 29484500 PMC5826914

[B37] Wang Y. Zhu D. Xu Y. Chen H. Zhang Y. (2019). Effects of seeding rate on seedling growth of machine-transplanted single-cropping hybrid rice. Hybrid Rice 34, 32–35. doi: 10.16267/j.cnki.1005-3956.20181119.298

[B38] Wu W. Nie L. Liao Y. Shah F. Cui K. Wang Q.. (2013). Toward yield improvement of early-season rice: other options under double rice-cropping system in central China. Eur. J. Agron. 45, 75–86. doi: 10.1016/j.eja.2012.10.009

[B39] Xiong D. Yu T. Zhang T. Li Y. Peng S. Huang J. (2015). Leaf hydraulic conductance is coordinated with leaf morpho-anatomical traits and nitrogen status in the genus oryza. J. Exp. Bot. 66, 741–748. doi: 10.1093/jxb/eru434 25429002 PMC4321541

[B40] Xiong Y. Wenyu Y. Wanjun R. (2009). Effects seedling raising methods and sowing rates on machine-transplanted long-age rice seedling. Trans. Chin. Soc. Agric. Eng. 25, 152–157. doi: 10.3969/j.issn.1002-6819.2009.06.029

[B41] Yang D. Cai T. Luo Y. Wang Z. (2019). Optimizing plant density and nitrogen application to manipulate tiller growth and increase grain yield and nitrogen-use efficiency in winter wheat. Peerj 7, e6484. doi: 10.7717/peerj.6484 30828492 PMC6396748

[B42] Ye H. Meng Y. Tang L. Zhu Y. Cao W. (2008). A simulation study on leaf age and leaf area index in rice. Chin. J. Rice Sci. 22, 625–630. doi: 10.3321/j.issn:1001-7216.2008.06.011

[B43] Yuan J. Liu Y. Xu K. Li G. Chen T. Zhou H.. (2022). Nitrogen and density treatment to improve resource utilization and yield in late sowing japonica rice. Acta Agronomica Sin. 48, 667–681. doi: 10.3724/SP.J.1006.2022.12018

[B44] Zhang M. Li Z. Feng K. Ji Y. Xu Y. Tu D.. (2023). Strategies for indica rice adapted to high-temperature stress in the middle and lower reaches of the yangtze river. Front. Plant Sci. 13. doi: 10.3389/fpls.2022.1081807 PMC9852850 36684799

[B45] Zhang X. Ding J. Liu Y. Gu Y. Han K. Wu L. (2014). Effects of mechanical transplanting of rice with controlled release bulk blending fertilizer on rice yield and soil fertility. Chin. J. Appl. Ecol. 25, 783–789. 24984497

[B46] Zhang Z. Wang J. Lang Y. Yu L. Xue Y. Zhu Q. (2008). Growing characteristics of rice seedlings of over-optimum age for mechanical transplanting. Acta Agronomica Sin. 34, 297–304. doi: 10.3724/SP.J.1006.2008.00297

[B47] Zhou L. Wu J. Gong K. Zhou H. Lu C. Huo Z. (2018). Research progress of machine-transplanted rice and substrate seedling raising techniques. China Rice 24, 20–23. doi: 10.3969/j.issn.1006-8082.2018.01.005

